# Synergistic Effects of Cabozantinib and EGFR-Specific CAR-NK-92 Cells in Renal Cell Carcinoma

**DOI:** 10.1155/2017/6915912

**Published:** 2017-12-20

**Authors:** Qing Zhang, Kang Tian, Jinjing Xu, Haixu Zhang, Liantao Li, Qiang Fu, Dafei Chai, Huizhong Li, Junnian Zheng

**Affiliations:** ^1^Cancer Institute, Xuzhou Medical University, Xuzhou, Jiangsu 221002, China; ^2^Department of Immunology, Binzhou Medical University, Yantai, Shandong 264003, China; ^3^Jiangsu Center for the Collaboration and Innovation of Cancer Biotherapy, Cancer Institute, Xuzhou Medical College, Xuzhou, Jiangsu 221002, China

## Abstract

The chimeric antigen receptor-modified immune effector cell (CAR-T and CAR-NK) therapies are newly developed adoptive treatments of cancers. However, their therapeutic efficacy against solid tumors is limited. Combining CAR-T or CAR-NK cells with chemotherapeutic drugs to treat solid tumor may be a promising strategy. We developed an epidermal growth factor- (EGFR-) specific third-generation CAR. NK-92 cells were modified with the CAR by lentivirus infection. The specific killing ability of the CAR-modified NK-92 cells (CAR-NK-92) against renal cell carcinoma (RCC) cell lines was confirmed *in vitro*. The synergistic effects of cabozantinib and EGFR-specific CAR-NK-92 cells were investigated *in vitro* and *in vivo*. Our results showed that the CAR-NK-92 cells lyse RCC cells in an EGFR-specific manner. Treatment with cabozantinib could increase EGFR and decrease PD-L1 membrane surface expression in RCC cells and enhance the killing ability of CAR-NK-92 cells against the RCC cells *in vitro*. Furthermore, the CAR-NK-92 cells show synergistic therapeutic efficacy with cabozantinib against human RCC xenograft models. Our results provided the basis for combination with chemotherapy as a novel strategy for enhancing the therapeutic efficacy of CAR-modified immune effector cells for solid tumors.

## 1. Introduction

Renal cell carcinoma (RCC) remains one of the most lethal urological cancers. Metastasis and recurrence occur in 20–30% of patients that received radical resection. It is also not sensitive to conventional radiotherapy and chemotherapy [1]. In recent years, small-molecule targeted therapy, including tyrosine kinase inhibitors (TKI), became the first-line treatment for metastatic RCC, though prognosis remains poor [2]. Immunotherapy is an exciting treatment option for RCC in the past decade. The most common immunotherapy includes cytokine therapy and immune checkpoint inhibition [3]. The use of cytokine therapy such as IL-2 and IFN-*α* declined because of modest response rates and poor tolerability. Immune checkpoint inhibitors have made significant progress and gained much attention with their approval for use in various solid tumors including RCC. However, their objective response rates are only 15–35% [4]. Therefore, it is urgent to develop new strategies to treat RCC.

The chimeric antigen receptor-modified T cell (CAR-T) therapy is a newly developed adoptive treatment of cancer. CAR-T therapy has achieved a gratifying breakthrough in hematological malignancies and showed exciting efficacy in some solid tumors, such as metastatic neuroblastoma [5, 6], recurrent glioblastoma [7], and prostate cancer [8]. However, its therapeutic efficacy in other solid tumors including RCC is less impressive. Lamers et al. designed a first-generation CAR (scFv-FcR*γ*) directed against carboxy anhydrase IX (CAIX) and used the CAR-modified T cells to treat patients with CAIX-expressing metastatic RCC [9]. Although blood cytokine profiles mirrored CAR-T cell presence and *in vivo* activity, no clinical objective responses have been observed in all of 12 patients.

Despite CAR-T therapy showed exciting efficacy in some cancers, the cost and severe toxicity (such as cytokine storm) have hindered its widespread use. Nature killer (NK) cell is another kind of immune effect cell contributing to the body's immune defenses. The unique biology of NK cells allows them to serve as a safe, effective, alternative immunotherapeutic strategy to CAR-modified T cells in the clinic [10]. NK cells can respond rapidly to transformed cancer cells and have the intrinsic potential to extravasate and reach their targets in tumor tissue. In addition to primary NK cells, also the established NK cell line NK-92 is being developed for adoptive immunotherapy. The NK-92 cell line was established from a 50-year-old male patient with rapidly progressive non-Hodgkin's lymphoma by Gong et al. and displays characteristics of activated NK cells [11]. General safety of infused NK-92 cells has been established in phase I clinical trials with clinical response observed in some treated renal cancer patients [12]. To enhance their therapeutic efficacy, NK-92 cells have been modified to express chimeric antigen receptors against different cancer targets, such as CD20 for lymphoma and leukemia [13], CD19 for chronic lymphocytic leukemia (CLL) [14], GD2 for neuroblastoma [15], EpCAM for breast carcinoma [16], Her2 for breast carcinoma and glioblastoma [17, 18], CS1 and CD138 for multiple myeloma [19, 20], EGFR for glioblastoma [21, 22], and CD3 or CD5 for T cell malignancies [23, 24]. The therapeutic efficacy of a combinational therapy of EGFR-CAR-modified NK-92 cells and oncolytic herpes simplex virus 1 was also tested in a mouse model with breast cancer brain metastases [25].

The hostile microenvironment composed of immunosuppressive cells (MDSC, Treg, macrophage, etc.) and molecules (TGF-*β*, PD-L1, PD-L2, etc.) is one of the most important factors that limit the therapeutic efficacy of CAR-modified immune effector cells against solid tumors [26]. The mechanisms of immune suppression of these factors have been well defined in previous reviews [27–29]. Elimination or inhibition of these immunosuppressive factors will significantly promote antitumor immunity and enhances the response to CAR-modified immune effector cell therapy [30]. A number of recent studies have indicated that besides their direct tumoricidal activity, some tyrosine kinase inhibitors (TKIs) can also modulate the tumor microenvironment and promote antitumor immunity. Doxorubicin [31, 32], sunitinib [33, 34], sorafenib [35, 36], and gemcitabine [37–39] have been proved to remodel the immune suppressive microenvironment and enhance antitumor immune response. They can augment the therapeutic efficacy of immunotherapy through combined application with them [32, 37, 40–42].

Cabozantinib is a TKI that was approved by the FDA in April 2016 for the treatment of advanced RCC [43]. It has been proved to increase the frequency of CD8^+^ and CD4^+^ T cells in the spleen and decrease the tumor infiltration of negative regulatory cell subsets, including MDSCs and Tregs [44]. Recently, cabozantinib was also reported to eradicate advanced prostate cancer by triggering a neutrophil-mediated anticancer innate immune response in a mouse model [45]. Furthermore, cabozantinib shows robust synergistic responses when combined with immune checkpoint blockade and cancer vaccine therapy by eliminating MDSC in tumor microenvironment in a mouse model [44, 46]. Therefore, cabozantinib can be used in a synergistic way to enhance the therapeutic efficacy of immune-based therapies for solid tumors.

In this study, we constructed a third-generation CAR against wild-type EGFR-positive cancers and sought to investigate synergistic therapeutic efficacy of the CAR-modified NK-92 cells combined with cabozantinib in a mouse model of human RCC.

## 2. Materials and Methods

### 2.1. Cell Lines

Human renal cancer cell lines (786-O, ACHN, Ketr-3, and OSRC-2) and colorectal cancer cell lines (HT29 and SW620) were obtained from Jiangsu Cancer Biotherapy Institute, Xuzhou Medical University, China. NK-92 cells were purchased from American Type Culture Collection (ATCC). The 786-O, OSRC-2, and HT29 cell lines were cultured in RPMI-1640 medium (Gibco, Life Technologies, America) supplemented with 10% FBS and 1% penicillin/streptomycin (Gibco, Life Technologies, America). The ACHN, Ketr-3, and SW620 cell lines were maintained in Dulbecco's modifed Eagle's medium (DMEM, Gibco, Life Technologies, America) supplemented with 10% FBS and 1% penicillin/streptomycin. NK-92 and transduced NK-92 cells were incubated in alpha modification of Eagle's minimum essential medium (*α*-MEM, Gibco, Life Technologies, America) supplemented with 2 mM L-glutamine, 0.2 mM myo-inositol, 0.02 mM folic acid, 0.1 mM 2-mercaptoethanol, 400 IU/ml IL-2 (Peprotech, America), 12.5% FBS and 12.5% horse serum (Gibco, Life Technologies, America), and 1% penicillin/streptomycin. All cell lines were cultured at 37°C in a humidified atmosphere with 5% CO_2_.

### 2.2. Flow Cytometric Analysis

For analysis of lentivirus transduction rate of NK-92 cells, the GFP expression in Ctrl-NK-92 and CAR-NK-92 was analyzed by a FACS machine (FACSCanto II, Becton-Dickinson, USA). For analysis of EGFR and PD-L1 surface expression, 1 × 10^6^ cancer cells were incubated with influorescence-labeled antibody in 200 *μ*l phosphate-buffered saline (PBS) with 2% bovine serum albumin (BSA) for 30 min at room temperature in dark, washed, and then analyzed by the FACS machine (FACSCanto II, Becton-Dickinson, USA). The PE-labeled mouse anti-human EGFR antibody (555997) and isotype control (555743) were purchased from BD Bioscience. The PE/Cy7-labeled mouse anti-human PD-L1 (329718) and isotype control (400325) were purchased from BioLegend.

### 2.3. Western Blot Analysis

Whole-cell lysates were prepared with RIPA buffer containing protease and phosphatase inhibitors. Equal amounts of cell lysates (25 *μ*g) were loaded on 10% SDS-PAGE and transferred onto pure nitrocellulose blotting membrane (Amersham, Sweden). After membranes were blocked, they were incubated with a primary rabbit anti-human anti-CD3*ζ* antibody (1 : 1000; ab40804, Abcam) or rabbit anti-human GAPDH antibody (1 : 1000; GTX100118, GeneTex). The membranes were then incubated with a horseradish peroxidase-conjugated anti-rabbit IgG. Target proteins were detected by the ECL system (Millipore) and visualized with the ChemiDoc XRS system (Bio-Rad).

### 2.4. ELISA Analysis

1 × 10^4^ target cells were cocultured with effector cells at effector cell : target cell (E/T) ratio of 0.5 : 1, 1 : 1, and 2 : 1 in round-bottom 96-well culture plates for 24 h, respectively. Cell-free supernatants were assayed for cytokine secretion by enzyme-linked immunosorbent assay (ELISA) kits according to the manufacturer's protocol. Human IFN-*γ* and perforin ELISA kits were purchased from Dakewe Biotech Company. Human granzyme B ELISA kits were purchased from BioLegend.

### 2.5. Cytotoxicity Assay

1 × 10^4^ target cells were cocultured with CAR-NK-92 or Ctrl-NK-92 cells at E/T ratio of 1 : 1, 3 : 1, 10 : 1, or 30 : 1 in RPMI-1640 with 15 mM HEPES and 5% FBS for 4 h. Released lactate dehydrogenase (LDH) in supernatants was measured using CytoTox 96 Nonradioactive Cytotoxicity Assay Kit (Promega, Madison, WI, USA) according to the manufacturer's instructions. Specific cytotoxicity was calculated according to the formula: % cytotoxicity = 100 × [(experimental release − effector spontaneous release − target spontaneous release)/(target maximal release − target spontaneous release)].

### 2.6. Cell Counting Kit-8 (CCK-8) Assay

The CCK-8 detection kit (Sigma-Aldrich) was used to measure cabozantinib cytotoxicity according to the manufacturer's instructions. Briefly, cells were seeded in a 96-well microplate at a density of 5000 cells and treated with DMSO or 2.5 *μ*g/ml cabozantinib for 0, 24, 48, 72, and 96 h. Subsequently, CCK-8 solution (10 *μ*l/well) was added and the plate was incubated at 37°C for 1 h. The viable cells were counted by absorbance measurements with a monochromator microplate reader at a wavelength of 450 nm. The optical density value was reported as the percentage of cell viability in relation to the control group (set as 100%).

### 2.7. In Vivo Efficacy Studies

5-week-old female Nod/Scid mice were purchased from Beijing HuaFuKang Biotechnology Co. Ltd. The mice were acclimated for 1 week in our animal facility before testing was initiated. The local committee for animal care approved all animal studies. A total of 5 × 10^6^ 786-O or ACHN cells were suspended in 100 *μ*l of RPMI 1640 or DMEM medium without FBS and penicillin/streptomycin and subcutaneously injected into the right flank of the mice, respectively. 5 days later (day 5), mice were randomly assigned to 5 treatment groups: untreated, cabozantinib (10 mg/kg), Ctrl-NK-92, CAR-NK-92, and combination of cabozantinib (10 mg/kg) and CAR-NK-92 (cabozantinib + CAR-NK-92). Mice of the cabozantinib group and the CAR-NK-92 + cabozantinib group were treated with cabozantinib by gavage on days 5–51 for 786-O cancer model and days 5–48 for ACHN cancer model, five times a week. From day 6 on, mice of the Ctrl-NK-92, CAR-NK-92, and CAR-NK-92 + cabozantinib groups received 3 × 10^6^ NK cells one time per week, 6 times in total. Meanwhile, the mice received NK cell therapy and received 2000 IU recombinant human IL-2 (rhIL-2) by intraperitoneal injection one time every two days. The length and width of the tumor were measured using a digital caliper, and the volume of the tumor was calculated using the formula: tumor volume = length × width^2^/2. Body weights of the mice were also recorded during the treatment. At the end of the experiment, tumor size was also monitored by bioluminescent imaging (BLI), then the mice were sacrificed and tumors were harvested for histologic analyses.

### 2.8. Immunohistochemistry

The harvested tumors were fixed in 10% neutral-buffered formalin, embedded in paraffin, and cut into 3–5 *μ*m sections. NK-92 cells in tumors were detected by immunohistochemistry staining using a rabbit anti-human CD3 antibody (ab40804, Abcam) at a 1 : 200 dilution. For the quantification of NK-92 cells in the tumors, the stained cells were counted in 10 randomly selected intratumoral fields of each slide under ×200 magnification.

### 2.9. Statistical Analysis

The data were analyzed using GraphPad Prism 5 software and presented as mean ± SEM. Statistical differences between the results of two groups were evaluated using two-tailed Student's test. The differences with *P* < 0.05 were considered statistically significant.

## 3. Results

### 3.1. Preparation and Characterization of Novel EGFR-Specific CAR-NK-92 Cells

**Figure 1:**
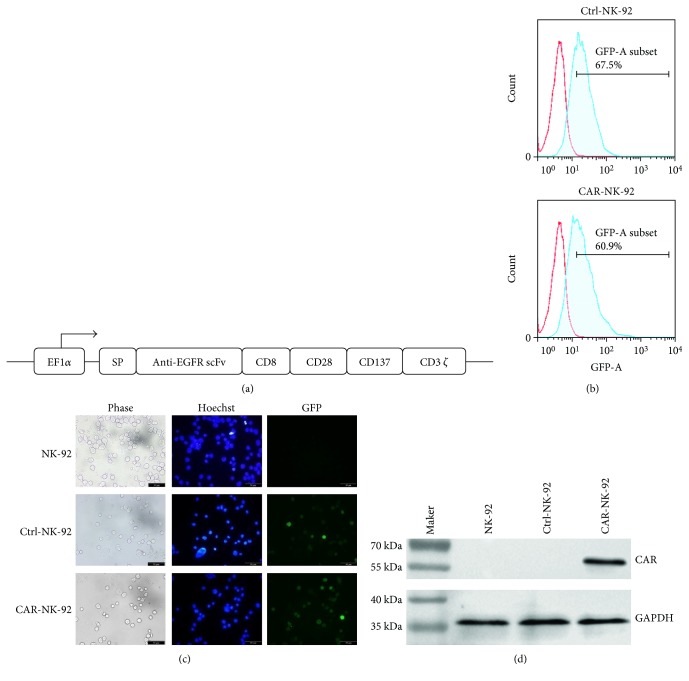
Generation and characterization of EGFR-specific CAR-NK-92 cells. (a) Structure diagram of EGFR-specific CAR. EF1*α*: promoter; SP: signal peptide; scFv: single-chain variable fragment. (b) Transduction efficiency of lentivirus in NK-92 cells. NK-92 cells were transduced with empty lentivirus vector (Ctrl-NK-92) or lentivirus containing the EGFR-specific CAR encoding sequence (CAR-NK-92) and selected with puromycin. The GFP expression was verified by FACS analysis. (c) GFP expression in lentivirus-transduced NK-92 cells in (b) was determined with a fluorescence microscope. The images were taken under ×400 magnification. (d) Western blotting analysis of the CAR expression in NK-92 cells with a monoclonal anti-human CD3*ζ* antibody. Glyceraldehyde-3-phosphate dehydrogenase (GAPDH) was also detected as an internal control.

A third-generation CAR, consisting of a wild-type EGFR-specific scFv linked to CD8 hinge and transmembrane domains and the intracellular signaling domains of CD28, 4-1BB, and CD3*ζ* in sequence (Figure 1(a)), was constructed and inserted into a lentiviral vector system with green fluorescence protein (GFP) and puromycin encoding sequences.

NK-92 cell line was transduced with the EGFR-specific CAR and empty lentiviral vector to generate CAR-NK-92 and Ctrl-NK-92 cells, respectively. As shown in Figures 1(b) and 1(c), following repeated selection of the transduced NK-92 cells with puromycin, the proportion of GFP-positive cells in both CAR- and empty vector-transduced NK-92 cells exceeded 60%. To validate the expression of EGFR-CAR in transduced NK-92 cells, we performed Western blot analysis using a rabbit anti-human CD3*ζ* monoclonal antibody that recognized the *ζ* chain portion of human CD3. The same as in previous report [13], the endogenous CD3*ζ* chains (~15 kDa) were detected in all NK-92 cells (data not shown). Whereas the EGFR-CAR was only detected in the CAR-transduced NK-92 cells (Figure 1(d)).

### 3.2. EGFR-Specific CAR-NK-92 Cells Specifically Kill EGFR^+^ Renal Cancer Cells In Vitro

**Figure 2:**
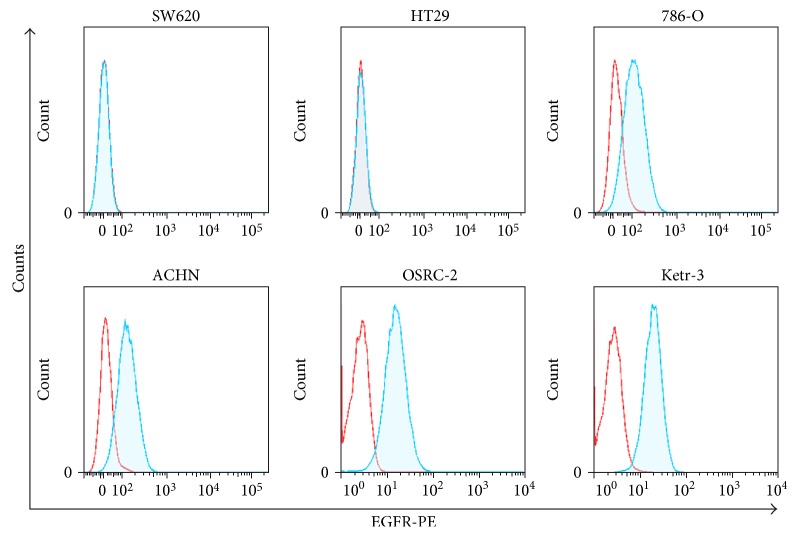
Surface expression of EGFR in human cancer cell lines. FACS was used to test the surface expression of EGFR proteins in human colon cancer SW620, HT29 cell lines, and renal cancer 786-O, ACHN, OSRC-2, and Ketr-3 cell lines.

FACS was used to assess the surface expression of EGFR in a series of human renal cancer cell lines, including 786-O, ACHN, OSRC-2, and Ketr-3. EGFR was strongly expressed in all renal cancer cell lines, with percentages ranging from 59% to 88.9% (Figure 2). Unfortunately, we did not find a renal cancer cell line that does not express EGFR. Therefore, we used EGFR-negative colon cancer cell lines SW620 and HT-29 as control target cells in the following experiments (Figure 2) [47, 48].

**Figure 3:**
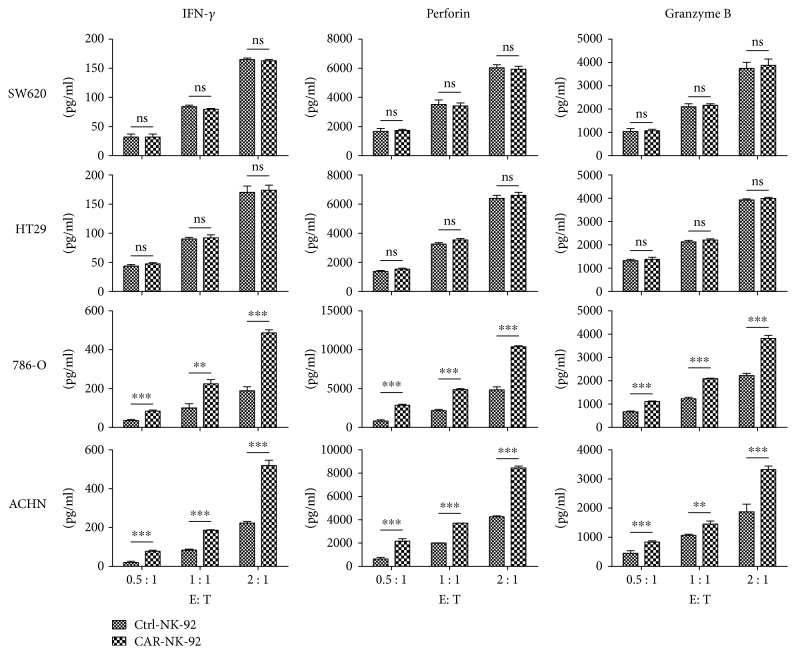
Specific cytokine release of EGFR-specific CAR-NK-92 cells against EGFR^+^ cells. The levels of cytokines, released by Ctrl-NK-92 and CAR-NK-92 cells, were measured by enzyme-linked immunosorbent assay (ELISA) after 24 h incubation with EGFR^−^ or EGFR^+^ target cells at an effector-to-target (E/T) ratio of 0.5 : 1, 1 : 1, and 2 : 1. ^∗∗^*p* < 0.01; ^∗∗∗^*p* < 0.001. ns: not significant.

To investigate whether the CAR-NK-92 cells could specifically recognize and be activated by EGFR-positive renal cancer cells, cytokine release assays were performed. The CAR-NK-92 and Ctrl-NK-92 cells were cocultured with cancer cells for 24 h at an effector-to-target (E/T) ratio of 0.5 : 1, 1 : 1, and 2 : 1, respectively. After incubation, the levels of cytokines released by CAR-NK-92 cells, including IFN-*γ*, perforin, and granzyme B, were significantly elevated in the supernatants of EGFR^+^ 786-O and ACHN cells compared with those of Ctrl-NK-92 cells. However, the levels of cytokines released by CAR-NK-92 cells and Ctrl-NK-92 cells were comparable when they were cocultured with EGFR-negative SW620 and HT29 cells (Figure 3). These results indicate that the CAR-NK-92 cells can specifically recognize and then be activated by renal cancer cells with high expression of EGFR.

**Figure 4:**
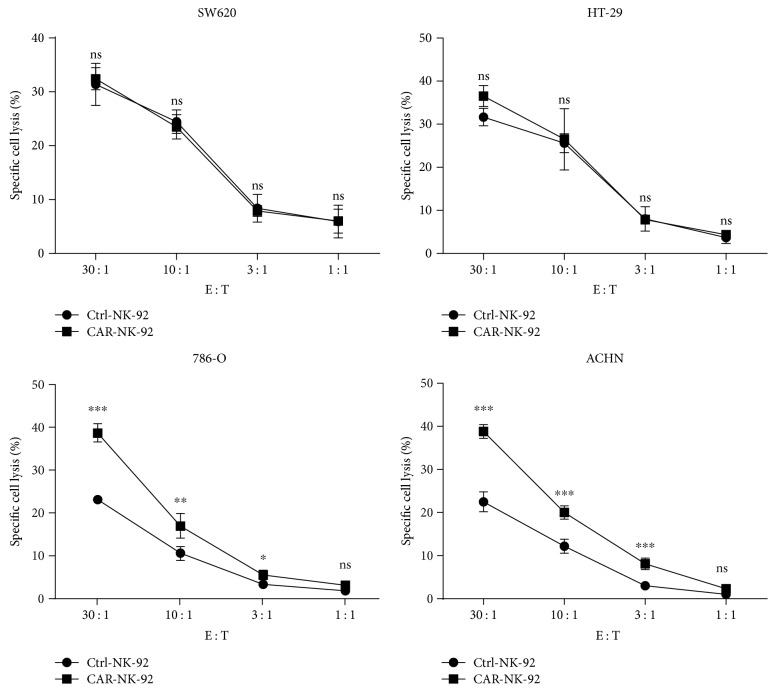
Specific cytotoxicity exhibited by EGFR-specific CAR-NK-92 cells against EGFR^+^ target cells. The cytotoxic activity of CAR-NK-92 and Ctrl-NK-92 cells against EGFR^−^ or EGFR^+^ cancer cells was determined using a 4 h lactate dehydrogenase (LDH) release assay in a dose-dependent manner. ^∗^*p* < 0.05; ^∗∗^*p* < 0.01; ^∗∗∗^*p* < 0.001. ns: not significant.

Next, to evaluate the cytotoxicity of the CAR-NK-92 cells against renal cancer cells, we performed dose-dependent lactate dehydrogenase (LDH) release assays. As shown in Figure 4, compared with Ctrl-NK-92 cells, CAR-NK-92 cells showed stronger killing activity against EGFR^+^ 786-O cells and ACHN cells at a ratio of 30 : 1, 10 : 1, and 3 : 1 E/T. However, the cytotoxicity difference between the two NK-92 cell lines against EGFR^−^ SW620 cells and HT29 cells was not significant. Additionally, the cytotoxicity of CAR-NK-92 cells against EGFR^+^ renal cancer cells was positively correlated with the E/T ratios. These results further demonstrated that the CAR-NK-92 cells could specifically recognize and kill EGFR^+^ renal cancer cells.

### 3.3. Impact of Cabozantinib on EGFR-Specific CAR-NK-92 Cells Function In Vitro

**Figure 5:**
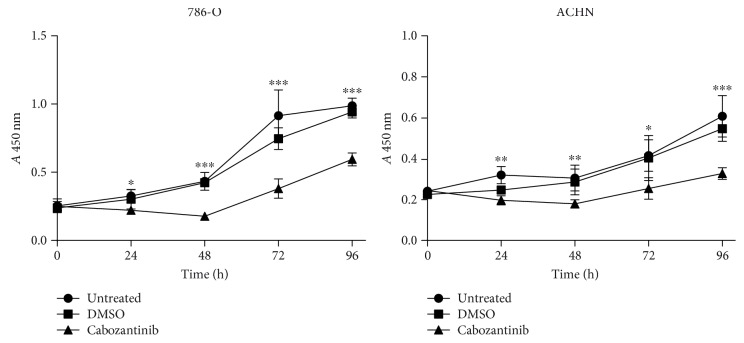
Growth inhibition of cabozantinib to renal cancer 786-O and ACHN cells. 786-O and ACHN cells were treated with 2.5 *μ*g/ml cabozantinib or vehicle (DMSO) for 1, 2, 3, and 4 days then assayed for growth and viability by CCK-8 assay. ^∗^*p* < 0.05; ^∗∗^*p* < 0.01; ^∗∗∗^*p* < 0.001.

To examine the *in vitro* immunomodulatory effects of cabozantinib, first, we determined the effect of cabozantinib on the proliferation of renal cancer cell lines 786-O and ACHN by cell counting kit-8 assay. 786-O and ACHN cells were exposed to 2.5 *μ*g/ml cabozantinib for 24, 48, 72, or 96 h to model the steady-state plasma concentration achievable in humans [44, 49]. As shown in Figure 5, cabozantinib significantly reduced the proliferation of 786-O and ACHN cells after 24, 48, 72, and 96 h. However, despite this reduction, the 786-O and ACHN cells continued to proliferate at all time points, regardless of treatment (Figure 5). We therefore used this dose of cabozantinib for all subsequent *in vitro* studies.

**Figure 6:**
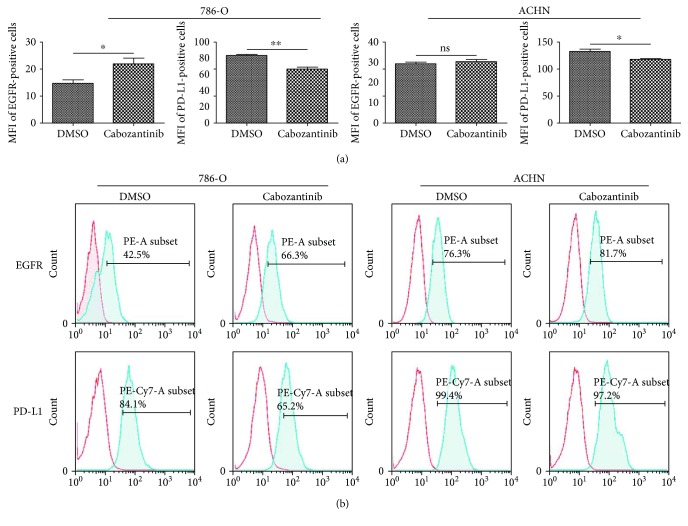
Cabozantinib alters the phenotype of renal cancer cells. 786-O and ACHN cells were exposed to 2.5 *μ*g/ml cabozantinib or vehicle for 24 h then analyzed by flow cytometry for surface expression of EGFR and PD-L1. (a) Representative figures from each group are shown, *n* = 3. (b) The corresponding quantitative analysis results of EGFR and PD-L1 expression in 786-O and ACHN cells shown in (a). ^∗^*p* < 0.05; ^∗∗^*p* < 0.01.

It has been previously shown that chemotherapy can alter the phenotype of tumor cells, rendering them more sensitive to NK cell-mediated killing [50, 51]. To determine if cabozantinib could modify the expression of cell-surface markers that influence the effect function of the EGFR-specific NK-92 cells, we treated 786-O cells and ACHN cells with cabozantinib for 24 h, then stained and analyzed them by flow cytometry. Cabozantinib treatment increased the percentage of 786-O and ACHN cells expressing EGFR that aid in the EGFR-specific CAR-NK-92 cell recognition and stimulation though the increasement was not statistically significant in ACHN cells. Cabozantinib also significantly decreased the expression of PD-L1 in 786-O and ACHN cells (Figure 6). The altered expression of these markers may make cancer cells more amenable to EGFR-specific CAR-NK-92 cell-mediated killing.

**Figure 7:**
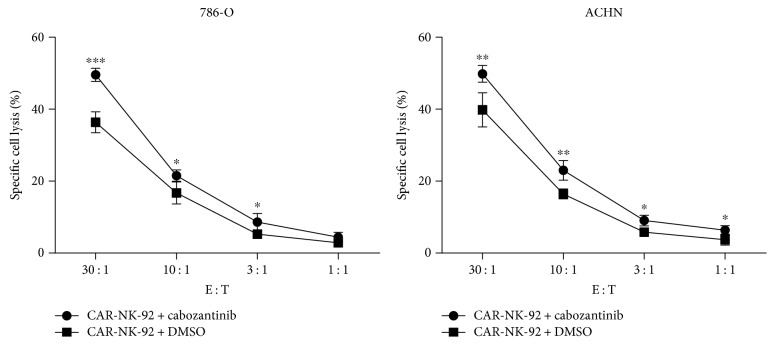
Treatment with cabozantinib increases the sensitivity of renal cancer cells to the CAR-NK-92 cell-mediated killing.786-O and ACHN cells were treated with 2.5 *μ*g/ml cabozantinib or vehicle for 24 h, then coincubated with the CAR-NK-92 cells for 4 h. The cytotoxic activity of CAR-NK-92 cells was determined by LDH release assay. ^∗^*p* < 0.05; ^∗∗^*p* < 0.01; ^∗∗∗^*p* < 0.001. ns: not significant.

To determine if cabozantinib treatment could increase the sensitivity of renal cancer cells to the CAR-NK-92 cell-mediated lysis, we treated 786-O and ACHN cells for 24 h, then used them as targets in LDH release assays. Cabozantinib treatment significantly increased the sensitivity of 786-O and ACHN cells to the EGFR-specific CAR-NK-92 cells (Figure 7). Taken together, these data suggested that cabozantinib was capable of altering renal cancer cells in ways that made them more amenable to the CAR-NK-92 cell-mediated attack and that cabozantinib and the CAR-NK-92 cells showed a synergistic killing effect on renal cancer cells *in vitro*.

### 3.4. Combination with Cabozantinib Further Improved the Antitumor Activity of EGFR-Specific CAR-NK92 Cells

**Figure 8:**
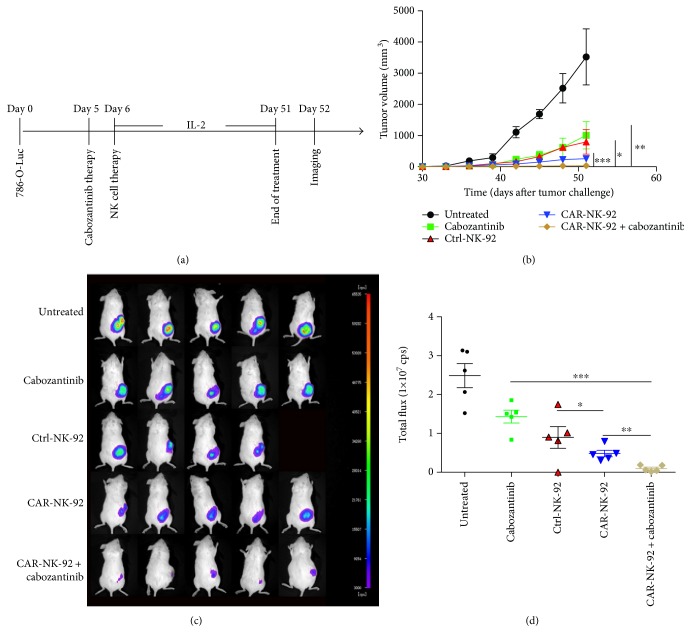
Therapeutic efficacy of EGFR-specific CAR-NK-92 cells combined with cabozantinib for human renal cancer xenografts established with 786-O cells. (a) Schematic diagram showing the treatment program of the mice. (b) The tumor growth curves during the experiment. (c) Luminescence images showing the tumor size at the end of the treatment. (d) Quantitative results of the tumor luminescence intensity shown in (c). ^∗^*p* < 0.05; ^∗∗^*p* < 0.01; ^∗∗∗^*p* < 0.001.

We evaluated the antitumor activity of Ctrl-NK-92 cells, CAR-NK-92 cells, cabozantinib, and CAR-NK-92 cells plus cabozantinib in NOD/scid mice with subcutaneous xenograft model established with 786-O cells expressing firefly luciferase (786-O-Luc). The treatment program of the mice was shown in Figure 8(a). Briefly, 5 × 10^6^ 786-O-Luc cells were subcutaneously injected into the right flank of the mice (day 0). Five days later (day 5), the mice of the cabozantinib and CAR-NK-92 + cabozantinib groups began to receive cabozantinib treatment for five times a week by gavage. One day later (day 6), the mice in the Ctrl-NK-92, CAR-NK-92, and CAR-NK-92 + cabozantinib groups began to receive 3 × 10^6^ Ctrl-NK-92 cell or CAR-NK-92 cell therapy, respectively, once a week, 6 times in total. From the day of NK-92 cell infusion, all mice were administered 2000 IU recombinant human IL-2 (rhIL-2) once every other day. All treatments ended on day 51. To monitor tumor growth, we measured the tumor dimensions using calipers during the treatment. On day 52, tumor sizes were also measured by *in vivo* imaging.

As shown in Figures 8(b)–8(d), Ctrl-NK-92 cells, CAR-NK-92 cells, or cabozantinib treatment alone significantly reduced the growth rate of 786-O-Luc tumors compared to the untreated group. In addition, treatment with EGFR-specific CAR-NK-92 cells significantly suppressed tumor growth compared with the Ctrl-NK-92 cells. This result demonstrated the EGFR-specific killing effect of the CAR-NK-92 cells to the 786-O-Luc tumors. Furthermore, the combination of the CAR-NK-92 cells and cabozantinib significantly reduced the growth rate of 786-O-Luc tumors compared to cabozantinib or CAR-NK-92 cell alone. The tumors of the mice that received CAR-NK-92 cells plus cabozantinib treatment were almost eradicated. The values of the tumor volumes were concordant with those of the *in vivo* imaging.

**Figure 9:**
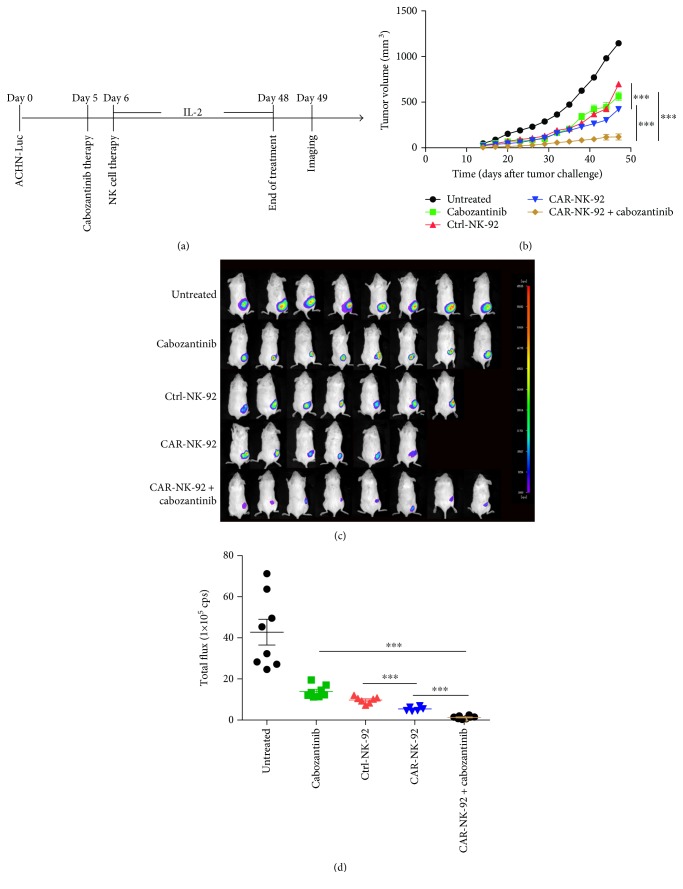
Therapeutic efficacy of EGFR-specific CAR-NK-92 cells combined with cabozantinib for human renal cancer xenografts established with ACHN cells. (a) Schematic diagram showing the treatment program of the mice. (b) The tumor growth curves during the experiment. (c) Luminescence images showing the tumor size at the end of the treatment. (d) Quantitative results of the tumor luminescence intensity shown in (c). ^∗∗∗^*p* < 0.001.

To further investigate the *in vivo* effect of the combination of EGFR-specific CAR-NK-92 cells and cabozantinib, we established another subcutaneous xenograft model in NOD/scid mice with human renal cancer ACHN cells expressing firefly luciferase (ACHN-Luc). The mice received the same treatment as that of the mice with 786-O-Luc tumors received (Figure 9(a)). As shown in Figures 9(b)–9(d), the rates of tumor growth were more considerably inhibited by treatment with EGFR-specific CAR-NK-92 cells than by that with Ctrl-NK-92 cells at the end of the treatment. In addition, the combination of the CAR-NK-92 cells and cabozantinib showed more significant antitumor effect than the CAR-NK-92 cells or cabozantinib alone in the ACHN-Luc xenograft model. Taken together, these data indicated that EGFR-specific CAR-NK-92 cells and cabozantinib have synergistic antitumor effects against EGFR-positive renal cancers.

### 3.5. The Homing Ability of EGFR-Specific CAR-NK-92 Cells

**Figure 10:**
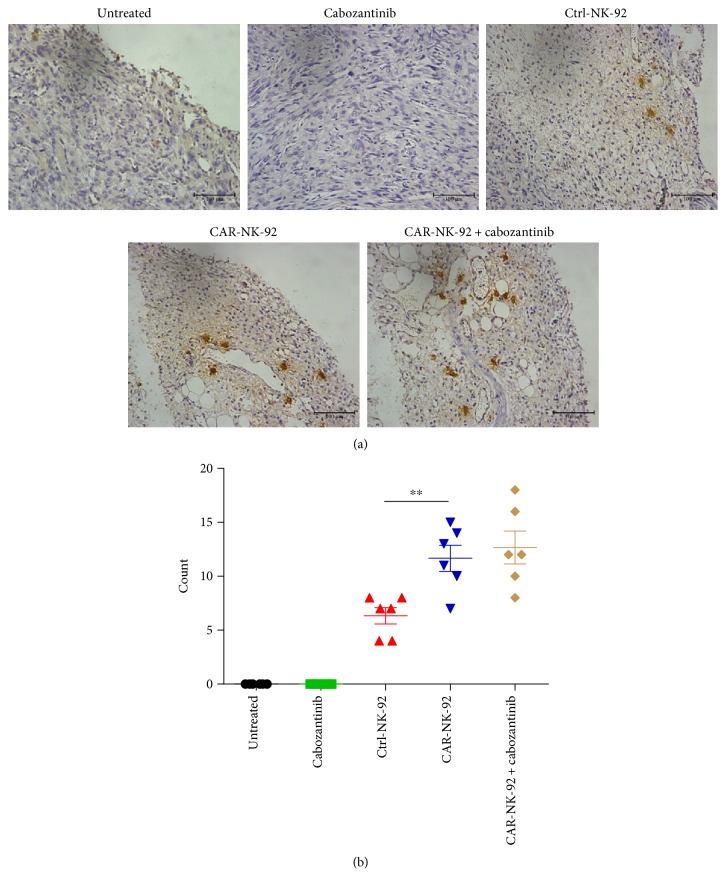
Tumor infiltration analysis of NK-92 cells *in vivo*. (a) Immunohistochemical analysis of human CD3^+^ NK-92 cells in established s.c. xenografts. The images were obtained under ×200 magnification. (b) The corresponding quantitative analysis results of human CD3^+^ NK-92 cells shown in (a). ^∗∗^*p* < 0.01.

To assess the homing ability of the CAR-NK-92 cells, immunohistochemical (IHC) staining with a monoclonal anti-human CD3*ζ* primary antibody was performed on tumor samples from the treated 786-O-Luc tumors described above. Both our Western blot data (not shown) and other's previous report [13] showed that wild-type NK-92 cells express CD3*ζ*. Therefore, both Ctrl-NK-92 cells and CAR-NK-92 cells homing into tumors could be detected by the IHC staining. As shown in Figure 10(a), no stained cell was observed in the tumor samples from the untreated and cabozantinib-treated mice. However, NK-92 cells were observed in the tumor tissue of the Ctrl-NK-92, CAR-NK-92, and CAR-NK-92 + cabozantinib groups though only a few positive cells could be seen in one visual field (Figure 10(a)). The statistic results showed that the number of NK-92 cells in the tumors of the CAR-NK-92 group was significantly higher than that in the tumors of the Ctrl-NK-92 group (Figure 10(b)). The number of NK-92 cells in the tumors of the CAR-NK-92 + cabozantinib group was higher than that in the tumors of the CAR-NK-92 group. However, the difference was not statistically significant. These findings suggest that the EGFR-specific CAR-NK-92 cells can effectively traffic to tumor sites.

## 4. Discussion

NK cells are vital immune effector cells and play important roles in immune surveillance. Numerous NK cell-based anticancer therapies are currently under investigation. However, therapeutic efficacies of the NK cell therapies in clinical trials to treat patients with solid tumors were very limited. This is largely due to the inability of NK cells to traffic into the tumor tissue and the immunosuppressive miroenvironment of the tumor. There are previous studies engineering NK-92 cells with chimeric antigen receptor to treat solid tumors. Here, for the first time, we reported that CAR-modified immune effector cell shows synergistic therapeutic efficacy with small molecular chemotherapy drug against solid tumor in preclinical tests.

MDSCs and Tregs are two immune subpopulations mediating immune suppression in the tumor microenvironment. Cabozantinib, a multikinase inhibitor, was reported to reduce the function of MDSCs and Tregs and show synergistic responses with immunotherapies against cancers [44, 46]. Based on these findings, cabozantinib may help the CAR-NK-92 cells to convert the immunosuppressive environment of tumors. In this study, we confirmed that treatment with cabozantinib could significantly increase EGFR expression in 786-O cell line and decrease PD-L1 expression in 786-O and ACHN cell lines. EGFR is the target antigen of the CAR-NK-92 cells. The increasement of EGFR may enhance the cytotoxic activity of the CAR-NK-92 cells to target cells. PD-L1 is a negative immunomodulatory ligand expressing on cancer cells, which binds PD-1 on immune effector cells (such as T cell and NK cell) to reduce their immune effect function. Furthermore, the decrease of PD-L1 expression may enhance the killing function of CAR-NK-92 cells to cancer cells. In addition to these findings, there may be other potential mechanisms for the synergistic efficacy that need to be further studied.

RCC is not sensitive to traditional radiotherapy and chemotherapy; however, it is sensitive to immunotherapy. Cytokines (IL-2 and IFN-*α*) and immune checkpoint inhibitors are the conventional immunotherapeutics used in clinic. Lymphokine-activated killer cell has been used to treat renal cancers in the eighties and nineties of the last century [52, 53]. Antigen-pulsed dendritic cells (DC) and genetically modified DC in combination with cytokine-induced killer cells (gmDC-CIK) were also reported for the treatment of RCC in the new century [54, 55]. Carbonic anhydrase IX- (CAIX-) specific CAR-T cell is one of the most closely watched cell therapies for RCC treatment [9, 56]. In a phase I/II trial with the CAR-T cells, 12 patients with CAIX+ mRCC were treated. The antigen-specific cytokine release of the CAR-T cells was detectable in all patients. Unfortunately, no clinical responses were observed. Moreover, CAR-T cell infusions induced liver enzyme disturbances reaching CTC (common toxicity criteria) grades 2–4 due to CAIX expression on bile duct epithelium in 4 patients [57]. To our knowledge, this is the first study investigating the potential of CAR-modified NK cells for RCC treatment.

For cabozantinib, previous studies showed that the clinical dose, 60 mg daily, showed significant toxicity in renal cancer patients [58]. Given a typical human weight of 60 kg, the clinical dose of 60 mg daily converting to a human dose is 1 mg per kg. To convert the human dose into mouse dose, we calculated 1 mg per kg × 12.3 = 12.3 mg per kg daily in mouse (the conversion factor 12.3 can be found in FDA guidance at http://www.fda.gov/downloads/Drugs/.../Guidances/UCM078932 and in [39]). The dose we used in the mice study, 10 mg per kg daily, is lower than the converted dose (12.3 mg per kg) from the clinical dose for RCC patients [46]. The results of in vivo study showed that treatment with the lower dose of cabozantinib showed synergistic responses with EGFR-specific CAR-NK-92 cells against RCC mouse model.

## 5. Conclusions

Our results show that EGFR-specific CAR-NK-92 cells have high potential to kill RCC cells, treatment with cabozantinib can increase EGFR and decrease PD-L1 membrane surface expression in RCC cells, and cabozantinib can enhance the effects of the CAR-NK-92 cells against RCC *in vitro* and *in vivo*. The current study is based on the NK-92 cell line adoptive therapy. Future study of this regimen can also be expanded to autologous or allogeneic primary NK or T cells. This study provides a novel strategy to enhance the therapeutic efficacy of CAR-modified immune effector cells for solid tumors.
